# Frailty and [^177^Lu]Lu-PSMA-617 therapy: tools and strategies for older men with metastatic castration-resistant prostate cancer

**DOI:** 10.7150/thno.131438

**Published:** 2026-07-29

**Authors:** Matteo Bauckneht, Nicolò Borsellino, Giuseppe Ferdinando Colloca, Lucia Fratino, Daniele Santini

**Affiliations:** 1 Department of Health Sciences (DISSAL), University of Genova, Genova, Italy.; 2 AOM-IRCCS Ospedale Policlinico San Martino, Genova, Italy.; 3 Medical Oncology Unit, Ospedale Buccheri La Ferla Fatebenefratelli, Palermo, Italy.; 4 Department of Geriatrics, Orthopaedics and Rheumatology, Fondazione A Gemelli IRCCS, Rome, Italy.; 5 Medical Oncology Unit, IRCCS Centro Di Riferimento Oncologico di Aviano, Aviano, Italy.; 6 Department of Sciences and Medical-surgical Biotechnologies, UOC Oncologia, Policlinico Umberto 1, La Sapienza Università di Roma, Rome, Italy.

## Abstract

The prognosis of metastatic castration-resistant prostate cancer is dismal. [^177^Lu]Lutetium-prostate-specific membrane antigen (PSMA)-617 ([^177^Lu]Lu-PSMA-617) is the new standard of care for PSMA-positive mCRPC previously treated with ARPI and taxane-based chemotherapy. The efficacy and safety of [^177^Lu]Lu-PSMA-617 have been confirmed by phase 2 (TheraP [NCT03392428]) and phase 3 (VISION study [NCT02511664] and PSMAfore trial [NCT04689828]) clinical trials. A substantial proportion of patients with mCRPC are older adults; however, chronological age alone should not be considered a contraindication to [^177^Lu]Lu-PSMA-617 therapy. Instead, therapeutic decisions must be guided by a comprehensive evaluation of life expectancy, comorbidities, and frailty. Here we review the effectiveness and safety data for [^177^Lu]Lu-PSMA-617, present tools for the geriatric and frailty assessments in clinical practice and discuss its utility and therapeutic indications in patients with different types of frailty.

## Introduction

Prostate cancer is an age-associated malignancy, with most diagnoses occurring in men older than 60 years. While many patients initially present with localised or castration-sensitive disease, a substantial proportion eventually develop metastatic castration-resistant prostate cancer (mCRPC), a condition associated with poor prognosis and limited long-term survival 1.

Several systemic therapies for mCRPC have demonstrated survival benefit, including taxane-based chemotherapy, androgen receptor pathway inhibitors (ARPIs), and poly(ADP-ribose) polymerase (PARP) inhibitors in selected molecular subgroups 2. However, in real-world clinical practice, a large proportion of patients with mCRPC are older, frail, and affected by multiple comorbidities 3-6. Because of this, treatment decisions are frequently limited by reduced physiological reserve, polypharmacy, impaired bone marrow function, and increased susceptibility to toxicity. As a result, standard systemic therapies are often poorly tolerated, leading to dose reductions, early discontinuation, or exclusion from treatment 7, 8.

In parallel, the role of geriatric oncology has shifted from selecting “fit” older adults for intensive treatments to enabling broader access to effective therapies through individualised, evidence-based decision-making. Comprehensive Geriatric Assessment (CGA) is no longer used primarily to exclude patients at high risk of toxicity, but rather to guide treatment adaptation and optimisation. This paradigm shift is particularly relevant in mCRPC, where frailty is highly prevalent and represents a central determinant of therapeutic sequencing 9-13. The goal today is to enable older adults, including those with degrees of frailty, to access life prolonging therapies through tailored, evidence-based decision making.

In this context, the limitations of conventional systemic therapies in frail patients highlight the need for alternative strategies. Prostate-specific membrane antigen (PSMA)-targeted radioligand therapy, particularly with [^177^Lu]Lu-PSMA-617, has emerged as an effective treatment option for PSMA-positive mCRPC 14, 15. By integrating molecular imaging for patient selection with targeted radionuclide delivery, this theranostic approach enables biologically driven, tumour-directed treatment. Although initially positioned after ARPIs and taxane-based chemotherapy, evolving regulatory approvals and accumulating clinical experience are progressively integrating radioligand therapy earlier in the treatment pathway, including in patients for whom chemotherapy is unsuitable 16-18.

Radioligand therapy should therefore not be regarded merely as a last-line option with uncertain feasibility in frail patients, but rather as a biologically rational and clinically meaningful strategy in a population where standard systemic treatments are frequently limited by toxicity. In this setting, the key issue is not whether radioligand therapy can be used in frail patients, but how to optimise patient selection and management to maximise benefit while minimising risk. Advanced age, impaired bone marrow reserve, baseline cytopenias, renal dysfunction, impaired nutritional status, and cumulative toxicity from prior treatments may increase vulnerability to treatment-related adverse events. Frailty should thus be considered not as a reason for exclusion, but as a factor requiring structured assessment, multidisciplinary evaluation, and individualised risk–benefit analysis 19.

Given the high prevalence of frailty in men with mCRPC 3-5 and the expanding role of PSMA-targeted theranostics, a critical appraisal of effectiveness, safety, and patient selection in this complex population is warranted. In this review, we examine the available evidence on [^177^Lu]Lu-PSMA-617 in frail patients, discuss tools for frailty assessment, and explore how radioligand therapy can be safely and appropriately integrated into therapeutic decision-making in mCRPC.

### [^177^Lu]Lu-PSMA-617

#### Efficacy/effectiveness

Lutetium-177 radioisotope, the active moiety of the drug, is linked to a small-molecule high-affinity PSMA-specific ligand. PSMA is a transmembrane protein highly expressed in prostate cancer, including the metastatic castration-resistant disease and its expression is lower in normal tissue than in tumour cells 14, 15. Once bound to PSMA-expressing cancer cells, the beta-emission of lutetium-177 induces DNA damage leading to cell death. [^177^Lu]Lu-PSMA-617 emits also a small fraction of gamma rays, which favours scintigraphic detection useful for post-treatment imaging 14.

The efficacy of the drug was first shown by the phase 3 VISION study (NCT02511664), in which [^177^Lu]Lu-PSMA-617 added to the standard of care prolonged radiographic progression-free survival (rPFS) and overall survival (OS) in patients with advanced PSMA-positive mCRPC 20. The magnitude of prostate-specific antigen (PSA) response was associated with clinical and patient-reported outcome improvements 21. Moreover, [^177^Lu]Lu-PSMA-617 plus standard of care delayed time to worsening of health-related quality of life and time to skeletal events compared to the control arm 22. The phase 2 TheraP (NCT03392428) trials showed that, compared with cabazitaxel, [^177^Lu]Lu-PSMA-617 elicited a better response measured as lowering of the PSA levels 23. Also, in a real-world setting, [^177^Lu]Lu-PSMA-617 provided significantly longer duration of PFS and OS 24 and of OS and time-to-treatment-failure as well as rates of at least 50% and 90% improvement of PSA levels versus baseline 25 than cabazitaxel. In addition, the PSMAfore trial (NCT04689828) showed that patients treated with [^177^Lu]Lu-PSMA-617 had twice longer median rPSF than those in whom ARPI was changed 26.

There is a growing body of evidence that confirms the effectiveness of [^177^Lu]Lu-PSMA-617 in the real-world setting 24, 27-32 and the results from a small series of 18 patients indicated that rechallenge with [^177^Lu]Lu-PSMA-617 radioligand therapy in mCRPC can be effective and safe 33. A meta-analysis of clinical trial results confirmed that the treatment with [^177^Lu]Lu-PSMA-617 favourably impacts the radiographic and biochemical control of mCRPC 34.

#### Safety

In the VISION trial, both all and grade ≥ 3 adverse events were more common in the [^177^Lu]Lu-PSMA-617 plus standard of care arm than in the control arm (52.7% versus 38.0%). Amongst adverse events of grade ≥ 3, haematological toxicity, namely anaemia (12.9% versus 4.9%), thrombocytopaenia (7.9% versus 1.0%), lymphopenia (7.8% versus 0.5%), was more common in the [^177^Lu]Lu-PSMA-617 plus standard of care arm 20. The mean haemoglobin and creatinine concentrations and platelet counts remained stable over time 22.

The VISION dosimetry substudy quantified absorbed doses of [^177^Lu]Lu-PSMA-617 in the kidneys and other organs showing that renal cumulative dose was below the established limit, whilst cumulative absorbed doses in at-risk organs over multiple cycles could be predicted from the first cycle data 35. In fact, according to a systematic review, tumour tissue receives 3-6 times greater absorbed dose than the at-risk organs 36.

The any-grade treatment-emergent adverse events (TEAE) frequency was similar across all cycles of [^177^Lu]Lu-PSMA-617 treatment. No additional safety concerns were reported for patients who received >4 cycles. The exposure-adjusted safety analysis revealed that the overall TEAE incidence was similar between arms, but distinct trends for different TEAE types were noted and the incidence of events associated with [^177^Lu]Lu-PSMA-617 remained higher in the [^177^Lu]Lu-PSMA-617 arm. Longer exposure to [^177^Lu]Lu-PSMA-617 plus standard of care was not associated with a higher toxicity risk, and the extended time for safety observation could account for the higher TEAE incidence in comparison to standard of care alone. The findings support a favourable benefit-risk profile for 6 cycles of [^177^Lu]Lu-PSMA-617 in this setting and the use of up to 6 cycles of [^177^Lu]Lu-PSMA-617 in patients who are clinically benefiting from and tolerating this therapy 37. For patients with metastatic prostate cancer no longer responding to hormone therapy, an increase in the number of cycles of treatment with [^177^Lu]Lu-PSMA-617 from 4 to 6 had no additional adverse side effects 37.

Regarding the risk of developing secondary tumours, a low incidence (5/381; 1.3%) of treatment-induced myeloid neoplasms was described after a median of 33.6 months from the commencement of [^177^Lu]Lu-PSMA-617 in patients who also underwent chemotherapy 38. A recent postmarketing safety monitoring study detected new adverse events (loss of libido, hydronephrosis, tachycardia, tumour lysis syndrome and tumour flare). These events were identified during a disproportionality analysis of the FDA Adverse Events Reporting System’s database and do not necessarily imply a causal relation to [^177^Lu]Lu-PSMA-617 39.

These findings highlight the need for meticulous patient selection and management to optimise therapeutic benefits while mitigating risks.

### Frailty

#### Definition and prevalence

The term “frailty” refers to a state of aging-related vulnerability due to physiological reserve and function decline. Such decline results in poor response to acute stressors increasing the likelihood of adverse events 40, 41. Several definitions of frailty have been proposed. Frailty can be conceptualised as the result of an accumulation of deficits such as diseases and disabilities that make an individual more vulnerable to health deterioration and increase the risk of complications and mortality 42, 43. The most widely used model of physical frailty describes it as a biological syndrome characterised by extreme vulnerability to stressors (physical, psychological or environmental) and predisposition to adverse events such as falls, disability, hospitalisation and death. It reflects a loss of homeostatic capacity and reduced resilience of biological system to environmental stress 6. According to Fried’s criteria, frailty can be defined as weight loss, exhaustion, low physical activity, slowness and weakness 40. In geriatric oncology, an early definition of frailty was proposed by Balducci et al, and included the presence of one or more of the following conditions: 1) a geriatric syndrome and/or 2) a dependency in at last one activity of daily living (ADL) and/or 3) more than three comorbidities and /or 4) age ≥ 85 years 44.

The weighted prevalence of frailty in community-dwelling >65-year-olds is about 10% and increases with age up to > 25% in the > 85 group 45. Given that > 80% of patients with prostate cancer receive their diagnosis at the age > 60 years, the problem of frailty is common in this patient category and impacts both the prognosis and therapy choices 19. Frailty measured by the Frailty Index (FI), based on the deficit accumulation model, is directly associated with worse baseline quality of life and impaired physical function in patients with mCRPC, underscoring its relevance not only as a prognostic variable but also as a practical tool for patient stratification before treatment decisions 46. This finding is consistent with data from the Italian real-world context: in the prospective Meet-URO 5-ADHERE study, a substantial proportion of patients with mCRPC presented with functional impairment on instrumental ADL assessment and G8 screening, confirming that frailty affects a clinically significant portion of this patient population already at the time of treatment planning 75.

#### Markers of frailty

Frailty is a multidimensional construct. It can be considered as an age-related, physical, clinical conditions, cognitive, psychological and social syndrome (Table 2) 47, 48. Frailty is considered to be an expression of population aging 6 and physical frailty is a pre-disability, disability being a condition in which assistance with basic ADL is needed 49. Low testosterone due to aging-related hypogonadism, as well as androgen deprivation therapy, a key of prostate cancer management, can cause sarcopenia, loss of bone mineral density, and increase the risk of falls and fatigue that contribute to frailty 3, 19.

Sarcopenia has garnered significant interest in oncology research and it is increasingly evident that reduced muscle mass and function are clearly associated with adverse outcomes and should be included in the geriatric assessment of older patient with cancer. According to the updated EWGSOP2 consensus, sarcopenia is defined by both low muscle strength and low muscle quantity or quality, with physical performance further stratifying its severity. In oncological practice, assessment of muscle mass via computed tomography at the L3 vertebral level or dual-energy X-ray absorptiometry is increasingly integrated into clinical workflows as an objective, reproducible measure of sarcopenic status that goes beyond the limitations of BMI or weight-based nutritional screening alone 50. In a secondary analysis of a prospective observational study in 110 men with mCRPC, baseline sarcopenia was a predictor of radiographic progression and overall mortality regardless of treatment type received 51. Cognitive frailty is the result of physical frailty and mild cognitive impairment and can be reversible or potentially reversible. Subjectively, patients may complain of memory loss 52. Psychological frailty has been defined as the co-occurrence of physical frailty with low mood, apathy, depression, loneliness, and cognitive deficits 48, 53. Social frailty, including living alone, interaction with neighbours, and social participation, is a component of frailty that increases the incidence of adverse health outcomes 54.

There is a relationship between frailty and poverty. The often-substantial financial burden can result in financial toxicity, i.e., unintended financial consequences and distress brought about by cancer and its treatment 55. Therefore, financial issues must be considered when discussing frailty 56. In an analysis from almost 500 older adults with cancer entered into the Cancer and Aging Resilience Evaluation (CARE) registry at the University of Alabama, the unmet resource needs were associated with a 3.3-fold higher adjusted odds of frailty. In this population, transportation insecurity (i.e., problems with getting transportation to the doctor and missing doctor’s appointments due to transportation issues) encountered in 11.5% was the most prevalent unmet need. Another CARE study showed that living in a rural area was associated with reduced 1-year survival amongst frail older adults with newly diagnosed cancer 57. Also, high prevalence and early onset of frailty was found in homeless individuals 58. Socioeconomic frailty results in health inequalities 59. In extreme situations, costs of healthcare may be so high that individuals delay or give up healthcare to pay for housing and food 60. In a Swedish study, patients with the highest incomes had higher probability of curative treatment for intermediate and high-risk prostate cancer (OR 1.77 [1.61-1.95]) 61. Finally, poverty increases social frailty of older individuals by disrupting their social activities, hindering building successful social relationships, and reducing quality of life 56.

#### Tools for measuring frailty

The primary goal of oncology treatment in frail patient includes symptom control, disease response or stability with acceptable toxicity coupled with the preservation of the quality of life.

**Figure 1:**
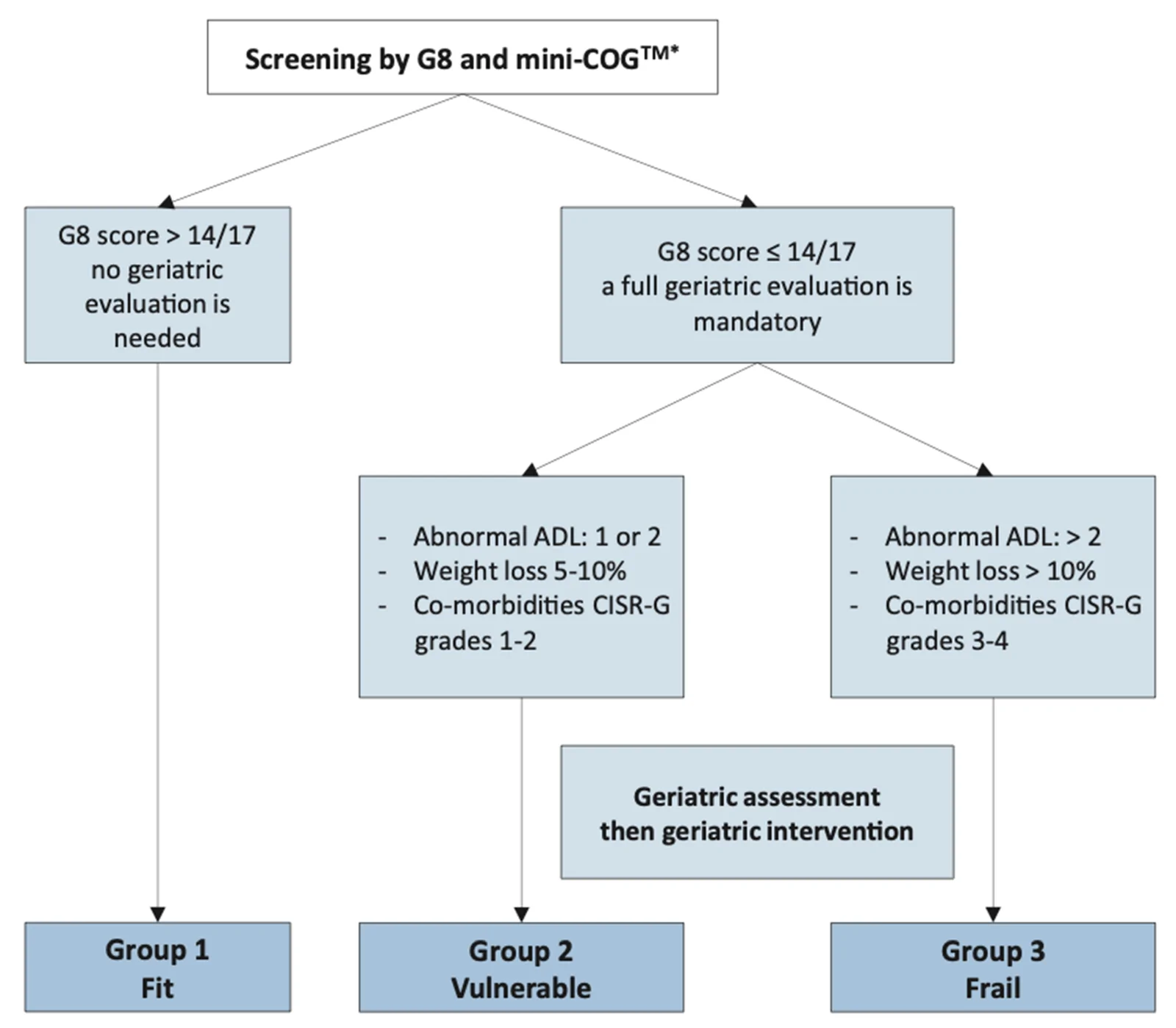
Health status screening recommended by the SIOG guidelines and included in the 2025 EAU guidelines on prostate cancer. Reproduced from EAU Guidelines. Edn. presented at the EAU Annual Congress Madrid 2025. ISBN 978-94-92671-29-5 originally published by and used with permission from EAU Guidelines Office, Arnhem, The Netherlands. http://uroweb.org/guidelines/compilations-of-all-guidelines/. Mini-COG^TM^, Mini- COG^TM^ cognitive test; ADLs, activities of daily living; CISR-G, cumulative illness rating score – geriatrics. *For Mini-COG^TM^, a cut-off points of ≤3/5 indicates a need to refer the patient for full evaluation of potential dementia.

EAU guidelines recommend the assessment of life expectancy and health status in patients with prostate cancer as the drivers of therapeutic decision-making. Full geriatric is not recommended in all patients (Figure 1) 62.

1. *Life expectancy*. Life expectancy can be predicted from gait speed usually measured over the distance of 6 meters: for men aged 75 years, 10-year survival ranged from 19% to 87% for gait speeds < 0.4 m/s and ≥ 1.4 m/s, respectively 63.

2. *Health status*. In oncology practice, abbreviated screening tools are frequently used to reduce assessment time. Geriatric 8 (G8) and Clinical Frailty Score (CFS) are comprehensive health status assessment tools 64-66. G8 discriminates between fit, vulnerable and frail patient based on the assessment of eight items: declining food intake, weight loss in 3 months, mobility, neuropsychological problems, BMI, polypharmacy (> 3 prescription drugs/day), self-comparison to same-age individuals, and age 66. The panel of the International Society of Geriatric Oncology (SIOG) proposed an operational flowchart, later incorporated into the EAU 2025 prostate cancer guidelines, for oncologists to incorporate geriatric screening using G8 and mini-COG™ into their standard clinical workflow (Figure 1). Patients with the G8 score ≤ 14 should undergo full comprehensive geriatric assessment (CGA) described below 62. In a Japanese study, 70% of patients with metastatic prostate cancer had G8 score < 14. The OS was significantly worse in patients with G8 ≤ 12 or > 12 and castration-resistant metastatic disease 67.

EAU guidelines list also the CFS tool, a nine-point scale (the higher the score the more pronounced frailty) less used in patients with cancer 41.

3. *CGA*. CGA is a multidimensional, often multidisciplinary, diagnostic process to assess functional status, comorbidities, cognition and emotional status, nutritional status, polypharmacy, and geriatric syndromes (fall risk, delirium, urinary incontinence, dentition, visual, or hearing impairments) 65. In patients with metastatic prostate cancer, CGA scores are lower than in patients without metastasis 4. The following tools can be used for a full CGA (Table 3):

a. Cumulative Illness Score Rating-Geriatrics (CIRS-G) and Charlson Comorbidity Index (CCI) are used to assess the burden of comorbidities 68, 69. The CIRS-G is a valid indicator of health status in the frail population, which performs well to predict longitudinal outcomes 69.

b. Body mass index or longitudinal monitoring of patient’s weight to assess nutritional status. By measuring weight for 3 months, malnutrition can be determined (good nutritional status: < 5% weight loss; risk of malnutrition: 5–10% weight loss; severe malnutrition: > 10% weight loss) 70.

c. Three-word recall and clock drawing (mini-COG™) are the tools used for cognitive function assessment 71. Mini-COG™ is a quick test to screen for cognitive impairments. Patients with scores ≤ 3/5 need full cognitive assessment and may be incapable of decision making 62.

d. Physical fitness is often evaluated using Karnofsky score and European Cooperative Oncology Group (ECOG) score. Karnofsky status is a 100-point scale that measures patient’s ability to perform various task. Increasing impairment results in lower scores 72. The ECOG performance score ranges from 0 (fully active) to 5 (dead) 73.

e. Patient’s independence assessment includes ADL. The index of ADL was developed to assess treatment results and prognosis in the elderly and chronically ill. Grades of the ADL evaluate the overall performance in bathing, dressing, going to toilet, transferring, continence, and feeding. The activities requiring higher cognition are assessed using instrumental ADL (IADL) score 74. In the population of patients with mCRPC enrolled into the Meet-URO 5-ADHERE study, instrumental ADL and the G8 geriatric scales were found to be essential tools to identify frail and less auto-sufficient patients who are vulnerable 75.

### [^177^Lu]Lu-PSMA-617 in frail and geriatric populations

The SIOG recommends that treatment in patients with prostate cancer should be based on patient’s health status evaluation and not on chronological age 76. Real-world studies show that age > 75 years, and even > 90 years, alone is not a contraindication for being treated with [^177^Lu]Lu-PSMA-617, as older patients had benefits when treated with this agent 77-83. The safety of [^177^Lu]Lu-PSMA-617 was confirmed in a real-life study of 37 patients > 75 years of age, meaning that advanced age in itself is not a contraindication for receiving this treatment modality 78. In fact, [^177^Lu]Lu-PSMA-617 treatment equally effectively controlled cancer and was safe in the elderly (> 75 years of age) and frail population 80, in two studies conducted on populations with a median age of 82 (range 75-92 and 80-92 years) at the start of the treatment 77, 79 and in a case series of three nonagenarians 82. Tauber et al showed clinical benefit with rare high-grade adverse events in a cohort of 80 octogenarians, especially those who were chemotherapy-naïve 79, whilst Bastian et al demonstrated that [^177^Lu]Lu-PSMA-617 in patients over 85 years (range 85-96 years), resulted in clinical and safety outcomes similar to those obtained in younger patients 81. This was further confirmed by a retrospective analysis of data from 102 patients with mCRPC in a single centre in Germany, which showed similar OS in patients < 80 and ≥ 80 with a comparable safety profile 83. Charron et al suggested that [^177^Lu]Lu-PSMA-617 could be a promising treatment option for medically-fragile nonagenarians with as the therapy elicited strong clinical and radiographic responses including significant PSA reductions. Its favourable safety profile and minimal toxicities further support its use in these medically-vulnerable patients 82. However, larger studies are needed to enable a more comprehensive analysis and establish the statistical significance of these finding.

Regarding frailty, a real-world multivariate analysis by Wenzel et al demonstrated that [^177^Lu]Lu-PSMA-617 was equally effective in frail patients (ECOG status ≥ 1) and in patients with ECOG status 0 despite worse PFS and OS in the frail population 80. Notably, the same analysis identified concomitant cardiovascular disease as an independent predictor of shorter PFS and OS 80. In addition, recent data from a cohort of 18 patients with poor performance status (ECOG 3) also showed that PSMA-targeted radioligand therapy is feasible in this patient category. In this cohort, 50% of patients achieved partial response or stable disease and severe adverse events (haematological toxicity) occurred in three patients 84.

At-risk organ-wise, despite haematological toxicity, the safety of [^177^Lu]Lu-PSMA-617 was confirmed in a cohort of 17 patients with preexisting moderate-to-severe thrombocytopaenia. There were no severe bleeds due to thrombocytopaenia and almost 60% of patients remained stable or showed improvement in severity of thrombocytopenia graded on Common Terminology Criteria of Adverse Events (CTCAE v5.0) severity score 85. One of the most frequent cause of reduced bone marrow reserve is diffuse bone marrow involvement. A multicentre retrospective study showed that the safety profile of [^177^Lu]Lu-PSMA-617 was acceptable in a study of 43 patients with diffuse bone marrow involvement, as grade ≥3 anaemia, thrombocytopaenia and neutropoenia were observed in 22%, 25% and 8% of patients, respectively 86. This datum was confirmed by Groener et al who showed that the cumulative treatment activity and absorbed whole-body dose did not correlate with new onset of grade ≥ 3 haematotoxicity in patients with diffuse bone marrow involvement 87.

**Figure 2:**
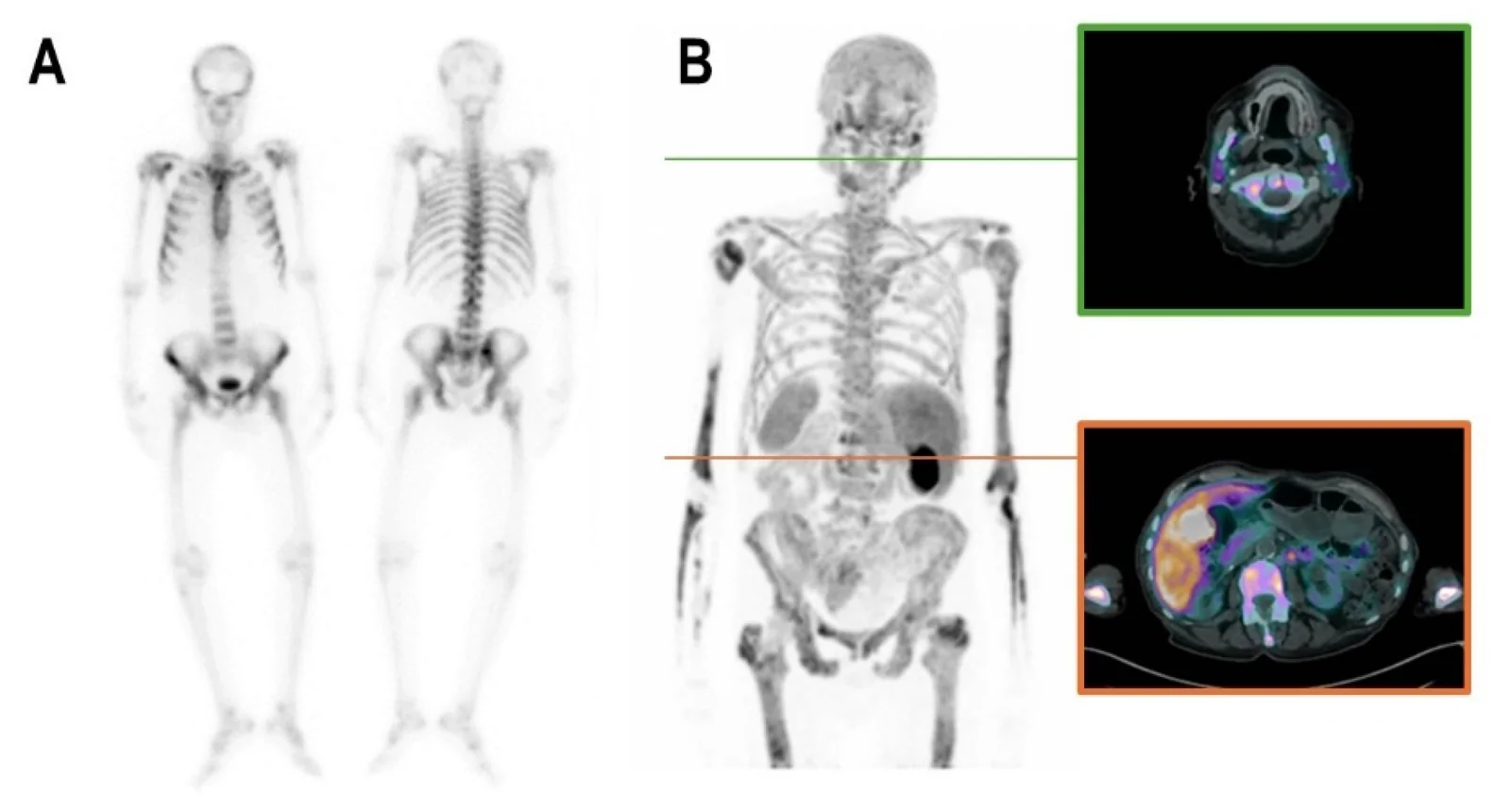
Understanding the “superscan” appearance in nuclear medicine on two emblematic examples of “superscan” patients. The “superscan” appearance on a bone scan (A) is characterised by diffuse, intense, and relatively symmetrical osseous radiotracer uptake, often accompanied by faint or absent tracer activity in the urinary system and soft tissues. Renal uptake is typically lower than that of the adjacent ribs, and bone metastases may not appear as distinct foci of metabolically active lesions 104. By contrast, the “superscan” pattern on PSMA PET imaging (B) is defined by diffusely increased PSMA uptake in PSMA-avid lesions, usually involving the entire skeleton, with minimal to no uptake in normal organs (salivary glands, intestine, spleen, and liver), as demonstrated in the hybrid PSMA PET/CT axial sections depicted on the right side of the panel. However, renal uptake was not suppressed in several cases described in the literature 105-108, and a consistent decrease in radiotracer uptake was not observed in all patients. Therefore, reduced renal uptake should not be considered a strict imaging criterion for the diagnosis of a PSMA PET “superscan”. Importantly, the presence of a “superscan” pattern on a bone scan does not necessarily imply a corresponding “superscan” pattern on PSMA PET imaging in the same patient.

Patients with diffuse marrow disease represent a unique challenge regarding radioligand therapy 88. Whilst not part of the VISION trial, a retrospective review of 43 patients from four institutions suggests that treating patients with diffuse marrow disease may be safe 86. SNMMI Consensus Statement on appropriate patient selection and use of [^177^Lu]Lu-PSMA-617 therapy noted that it is not well defined how to translate diffuse marrow disease on bone scan to PSMA PET. Overall, the committee agreed that patients with diffuse marrow disease are candidates for PSMA radioligand therapy 88. In nuclear medicine, the term “superscan” historically described patients with extremely high-volume bone metastases as visualised on bone scans (Figure 2 A). More recently, the concept has been extended to PSMA PET imaging (Figure 2 B). So far, [^177^Lu]Lu-PSMA-617 has not been extensively studied in patients with “superscan” bone scans, as the presence of the classical “superscan” in baseline bone scintigraphy was an exclusion criterion in clinical trials, including the VISION study. However, the PSMA PET “superscan” pattern was not an exclusion criterion, and such patients remain eligible for treatment (for further explanation please see the legend of Figure 2). Notably, real-world data indicate that patients with “superscan” pattern on bone scans may still respond well to therapy 86, 89-91. Altogether, these data suggest that while caution is warranted, treatment response appears to be independent of the number of bone metastases and total tumour burden. Therefore, PSMA-targeted radionuclide therapy can be considered safe even in patients with high disease volume.

Preexisting renal impairment was not associated with significantly poorer safety outcomes in real-world studies, although treatment led to declines in eGFR 92-94. In the Bastian et al study, CTCAE severity score of renal impairment remained stable in ~60% of patients 92-94, whereas in the Rosar et al analysis, renal function improved in 10/22 (45.5%), remained unchanged in 11/22 (50%) and worsened in just one patient 94. In the latter study, a statistically significant increase in cohort’s mean eGRF versus baseline was observed at the end-of-treatment (after a median of five cycles) and after 6 months of follow. Mean eGRF was still improved after 9 and 12 months of follow-up albeit the difference versus baseline was not statistically significant 94. In a German retrospective analysis of 106 patients (mean age 73 years), an at-least-moderate (>15%) eGFR decline was observed in 45% of patients who received four or more cycles of [^177^Lu]Lu-PSMA-I&T at 12 months after treatment commencement. In 24% (25/106) of patients, a severe eGFR (≥30%) decline was seen. A higher number of risk factors (including arterial hypertension, age ≥ 65 years, diabetes mellitus, prior platinum-based chemotherapy) at baseline was associated with a greater eGFR decrease at 12 months 95. A case report confirmed the safety and symptomatic improvements following treatment with [^177^Lu]Lu-PSMA-617 in a kidney transplant recipient 96. The overall safety in patients with moderate and severe renal impairment is specifically being analysed in an ongoing trial (NCT06004661) 97.

### Clinical experience and strategies to manage [^177^Lu]Lu-PSMA-617 therapy in frail patients

#### Multidisciplinary clinical experience in frail patients

The evolution of geriatric oncology has shifted the paradigm from binary “fit versus frail” classifications to a dynamic model of treatment adaptation based on biological age, functional reserve, and treatment-specific toxicity profiles 12, 76. Although this conceptual framework is supported by geriatric oncology principles rather than radioligand therapy-specific prospective data, it is highly relevant in the context of theranostic approaches, the treatment burden and administration logistics of which differ substantially from conventional systemic approaches. Geriatric oncology coined the concepts of “elderly-friendly” pharmacological profiles and “adapted schedules”, supporting a personalised approach to treatment selection 76.

These following expert-driven insights are based on our multidisciplinary clinical experience with treating frail patients who have mCRPC. Our team, including medical oncologists, nuclear medicine specialists, and geriatricians, finds that frailty alone seldom leads to an automatic refusal of PSMA-targeted radioligand therapy. Instead of serving as an exclusion criterion, frailty typically guides adjustments to treatment plans and determines how closely patients are monitored.

Five frequently encountered clinical scenarios include:

1. Chemotherapy-ineligible patients with PSMA-positive disease

A substantial proportion of older patients with mCRPC are deemed unsuitable for taxane-based chemotherapy due to reduced performance status (ECOG 1–2), relevant cardiovascular comorbidities, prior intolerance to systemic therapies, or borderline bone marrow reserve. In these individuals, treatment decisions are often limited, not by tumour biology, but by host vulnerability. In our experience, patients with PSMA-positive disease who are chemotherapy-ineligible may still tolerate radioligand therapy with manageable haematological toxicity, particularly when careful baseline assessment and early laboratory monitoring are implemented. This observation is consistent with emerging real-world evidence suggesting feasibility of radioligand therapy in elderly and clinically vulnerable populations 77-82, although prospective data specifically addressing this subgroup remain limited. Compared with cytotoxic chemotherapy, the intermittent administration schedule and tumour-targeted radiation delivery may offer a more acceptable therapeutic burden in selected frail individuals.

2. Patients with cardiovascular disease

The finding of cardiovascular disease being an independent predictor of shorter PFS and OS in patients treated with [^177^Lu]Lu-PSMA-617 80 has clinically relevant implications in this patient population. From a geriatric perspective, this observation is not unexpected: the burden of cardiovascular comorbidity is both highly prevalent and particularly difficult to quantify in patients aged over 75. As highlighted by current ESC guidelines, standard cardiovascular risk scores lose calibration in older adults and tend to underestimate true risk, especially in the presence of polypharmacy, frailty-related haemodynamic vulnerability, or subclinical organ dysfunction 98. The coexistence of cardiovascular disease and mCRPC therefore warrants structured cardiac evaluation as an integral component of the pre-treatment multidisciplinary geriatric assessment, rather than being considered merely as a background comorbidity.

3. Patients with cognitive vulnerability and treatment adherence problems

Mild cognitive impairment, frequently encountered in older adults with advanced cancer, should not be considered a contraindication to radioligand therapy. There is currently no prospective evidence specifically addressing the impact of cognitive impairment on radioligand therapy outcomes; however, this consideration arises from practical differences in treatment delivery of various therapeutic options. A continuous oral ARPI therapy requires sustained daily adherence, appropriate drug management, and monitoring for long-term metabolic and cardiovascular toxicity, which may be challenging in patients with cognitive vulnerability, polypharmacy, or limited caregiver support. By contrast, radioligand therapy delivered at fixed intervals in a controlled hospital setting allows structured supervision, scheduled assessment, and multidisciplinary follow-up. In selected cases, this model may therefore be operationally easier to manage than chronic oral systemic therapy, provided that adequate social and caregiver support is available.

4. Patients with baseline cytopaenias or diffuse bone involvement

Frailty in mCRPC is frequently associated with reduced bone marrow reserve, either due to prior therapies or extensive bone metastases. While significant baseline cytopenias warrant caution, we do not systematically exclude patients solely on this basis. Available retrospective data suggest that radioligand therapy may be feasible even in patients with compromised marrow reserve or diffuse bone involvement 85-87, although an increased risk of haematological toxicity should be expected. In selected cases, we adopt intensified laboratory monitoring during the first treatment cycles, provide transfusion support, when necessary, and perform early reassessment after the first cycle before proceeding further. This risk-adapted approach allows individualised treatment rather than a priori exclusion, particularly in patients with symptomatic high-volume disease and limited alternative options.

5. Patients with functional dependence and the role of the caregiver

Functional status, particularly as measured by IADL, may influence treatment feasibility in different ways depending on the therapeutic modality. There is currently no direct evidence comparing the impact of functional dependence on outcomes across different mCRPC treatments; however, practical considerations related to treatment administration are relevant.

- Oral therapies: in the context of oral agents (e.g., ARPIs), the presence of an active caregiver may compensate for reduced patient autonomy, supporting adherence and safe treatment administration.
- Radioligand therapy: conversely, radioligand therapy involves specific radioprotection measures following administration, requiring patients to comply with hygiene and safety recommendations.

In this setting, functional independence may become relevant for safe outpatient management, although this should not be interpreted as an absolute exclusion criterion. These considerations are therefore primarily logistical rather than biological and should be evaluated on a case-by-case basis 99.

Table 3 summarises the patient selection and suitability considerations. Our clinical experience suggests that frailty assessment should guide treatment adaptation, monitoring strategies, and supportive care planning, rather than function as an automatic exclusion criterion. Importantly, for several of the scenarios discussed, high-quality prospective evidence is currently lacking, and these considerations should therefore be interpreted as expert-based clinical reasoning aimed at supporting, rather than replacing, evidence-based decision-making. Future prospective studies specifically addressing frail populations are warranted to better define these aspects.

To integrate these complexities, we propose a formalised four-step clinical pathway:

1. Staging the Ageing: A multidisciplinary evaluation involving oncologists, geriatricians, and specialised nursing staff. Initial screening (e.g., G8 scale) identifies candidates who require a full CGA.

2. Prehabilitation: Proactive intervention to address malnutrition, pain, social isolation, and comorbid conditions prior to treatment initiation.

3. Toxicity Minimisation: Selecting the "right treatment for the right patient" with a primary focus on preserving quality of life and ensuring treatment completion.

4. Rehabilitation and Simultaneous Care: Integrating supportive care early in the trajectory to manage the treatment journey and facilitate recovery.

#### Monitoring and toxicity-adapted management during [177Lu]Lu-PSMA-617 therapy

Monitoring during [^177^Lu]Lu-PSMA-617 therapy is based on standardised laboratory, clinical, and imaging assessments as recommended by current EANM/SNMMI procedural guidelines 100. Laboratory tests should be obtained prior to each treatment cycle (within 5 days) and include full blood count, renal function (serum creatinine and estimated glomerular filtration rate), liver function parameters (AST, ALT, bilirubin, albumin, alkaline phosphatase), as well as disease-related markers such as PSA, LDH and CRP. During treatment, blood counts and renal function should be reassessed at least 2–3 weeks after each cycle (corresponding to the expected nadir) and again 4–6 weeks after completion of therapy. PSA should be monitored at each cycle and at follow-up, and interpreted according to PCWG3 criteria, considering the possibility of delayed response or PSA flare 100. Imaging-based assessment is typically performed using PSMA-PET/CT, or alternatively PSMA-SPECT/scintigraphy combined with morphological imaging, at intervals of approximately 12 weeks or at the end of a treatment series 100. Post-therapeutic scintigraphy may also be used to confirm biodistribution and provide an early qualitative assessment of treatment response. In addition, patient-reported outcomes, including fatigue, pain, and functional status, represent key elements in decisions regarding treatment continuation.

Data from the VISION trial indicate that the overall frequency of TEAEs remains relatively consistent across treatment cycles, without a clear cumulative increase in toxicity burden, although haematological toxicity typically occurs early, with nadirs observed during the first cycles 37. Real-world evidence from expanded-access programs additionally demonstrates that this safety profile is applicable to more clinically vulnerable populations, with similar rates of grade ≥3 anaemia, thrombocytopenia, and neutropenia despite worse baseline characteristics 28, 39, 83, 86, 87.

Importantly, monitoring findings should directly guide treatment adaptation. According to regulatory guidance and clinical trial evidence, the occurrence and severity of adverse events determine the need for treatment interruption, dose reduction, or discontinuation 101. Treatment should be withheld in the presence of clinically relevant toxicities, including haematological toxicity (e.g., grade ≥2 cytopenias), renal impairment, significant non-haematological adverse events (e.g., grade ≥3 fatigue or gastrointestinal toxicity), or clinical complications such as spinal cord compression or fractures in weight-bearing bones, until recovery to grade 1 or baseline. If treatment delay exceeds 4 weeks due to toxicity, permanent discontinuation is recommended.

Dose modification strategies are similarly toxicity driven. A single dose reduction of approximately 20% (to ~5.9 GBq) may be considered in selected cases, including severe xerostomia, gastrointestinal toxicity, clinically significant myelosuppression, or renal toxicity (e.g., marked increase in creatinine or decline in eGFR). Dose re-escalation is not recommended, and treatment should be discontinued if further dose reductions would be required 101. In the VISION trial, dose reductions were required in a minority of patients (5.7%), most commonly due to haematological toxicity, including thrombocytopaenia and anaemia 20. In a prospective Italian phase 2 study, [^177^Lu]Lu-PSMA-617 therapy at 5.5 GBq per cycle was safe and effective, and led to survival outcomes comparable to studies using 7.4 GBq with fewer severe toxicities supporting tailoring regimen intensity in patients at higher risk of toxicity 102.

Management of specific toxicities requires a risk-adapted approach 101. Haematologic toxicity typically warrants treatment delay until recovery to grade 1 or baseline, with supportive care measures including transfusion or growth factors when indicated, and dose reduction in the case of grade ≥3 events. Renal toxicity requires treatment interruption until improvement, with dose reduction or discontinuation in cases of persistent or severe impairment. Non-haematological toxicities—including gastrointestinal symptoms, fatigue, and metabolic abnormalities—should prompt treatment delay until recovery to grade ≤2 or baseline. Severe hepatic toxicity (e.g., AST/ALT >5× upper limit of normal in the absence of liver metastases) represents an indication for treatment discontinuation 101.

While these principles apply to all patients, frailty may influence both the intensity of monitoring and the thresholds for intervention. In frail individuals, particularly those with reduced bone marrow reserve, diffuse bone involvement, renal impairment, or high comorbidity burden, closer surveillance is advisable, especially during early treatment cycles. This may include earlier interim laboratory assessments (e.g., within 10–14 days), more frequent reassessment in patients with borderline baseline values, and proactive planning of supportive care interventions. In these patients, treatment adaptation may be considered at lower toxicity thresholds compared with non-frail patients, particularly when cumulative clinical burden (e.g., fatigue, functional decline, or borderline cytopenias) is observed. Conversely, selected frail patients may still benefit from continuation of therapy under intensified monitoring and multidisciplinary supportive care. Integration of geriatric assessment domains (functional, cognitive, and social factors) may further support individualised decision-making.

Overall, monitoring during [^177^Lu]Lu-PSMA-617 therapy should be interpreted as a dynamic process integrating toxicity detection, treatment adaptation, and supportive care, with a tailored approach in frail patients aimed at maintaining treatment feasibility while minimising clinical risk.

## Conclusions

In order, to maximise the outcomes, [¹⁷⁷Lu]Lu–PSMA-617 therapy must be offered to the appropriate patients meaning that careful patient selection is of utmost importance. Hence, prior to treatment initiation, patients must undergo a detailed assessment both to establish the eligibility and baseline key organ function for on-treatment comparisons. Equally important is treatment monitoring for early identification and management of adverse events. We propose a checklist that details the pre-, during, and post-treatment tests (Table 4).

In the future, the selection of the most suitable treatment for any given patient may be based on predictive circulating genomic markers. For example, in the TherP trial, patients with *ATM* gene mutations had favourable outcomes with [¹⁷⁷Lu]Lu–PSMA-617 therapy 103. Our review of the literature shows that [^177^Lu]Lu-PSMA-617 is a valid therapeutic option in patients with mCRPC considered frail due to age and comorbidities.

## Figures and Tables

**Table 1:** Dimensions of frailty. Adapted from 48

Dimension	Description
Physical	• Frailty syndrome: set of signs and symptoms that define a health condition • Age-related accumulation of diseases and deficits
Cognitive	• Reduced cognitive reserve, can be reversible or not
Social	• Risk of losing/loss of resources needed to fulfil basic social needs, threat to/lack of self-management abilities to fulfil social needs • Low income, housing insecurity
Psychological	• Co-occurrence of frailty with low mood, apathy, depression, loneliness and cognitive deficits.

**Table 2:** Tools used for assessing frailty.

Domain	Tools
Comprehensive health status	Geriatric 8 (G8), Clinical Frailty Score (CFS)
Comorbidities	Cumulative Illness Score Rating-Geriatrics (CISR-G) and Charlson Co-morbidity Index (CCI)
Nutritional status	BMI, longitudinal body weight measurements
Cognitive function	Mini-COG
Physical function	Performance status: Karnofsky score, ECOG score Activities of Daily Living (ADL) and Instrumental Activities of Daily Living (IADL)

ECOG, Eastern Cooperative Oncology Group.

**Table 3:** Patient selection and suitability considerations for [^177^Lu]Lu-PSMA-617 radioligand therapy.

Indications
EU: adults with progressive PSMA-positive metastatic castration-resistant prostate cancer previously treated with ≥1 ARPI and ≥1 taxane-based chemotherapy in combination with ADT ± AR pathway inhibition 101
US: adults with PSMA-positive metastatic castration-resistant prostate cancer who have been treated with ARPI and taxane-based chemotherapy **and** adults with PSMA-positive metastatic castration-resistant prostate cancer previously treated with ARPI in whom the delay of taxane-based chemotherapy is considered appropriate 16, 17
Clinical scenarios in which treatment may be considered (supported by available evidence and consensus statements):
• Patients unsuitable for taxane-based chemotherapy but with PSMA-positive disease 100 • High tumour burden or bulky disease 100 • Diffuse bone marrow disease or PSMA PET “superscan” pattern (with careful monitoring) 88, 91
Clinical scenarios requiring individualised multidisciplinary risk–benefit discussion (expert-based considerations; not absolute contraindications):*
• Reduced performance status (ECOG PS ≥2) • Significant baseline cytopenias or limited bone marrow reserve • Mild-to-moderate cognitive impairment • High comorbidity burden (e.g., elevated CIRS-G or CCI score) • Functional decline (ADL/IADL impairment) • Severe renal insufficiency • Severe hepatic dysfunction • Severe cardiopathy with risk of haemodynamic instability • Active uncontrolled infection • Limited social support or logistical barriers

* The clinical considerations listed here reflect expert-based multidisciplinary experience and should not be interpreted as formal contraindications. Frailty-related dimensions (functional, cognitive, social and comorbidity burden) should guide treatment adaptation, monitoring intensity and supportive care planning rather than automatic exclusion from radioligand therapy. ADT, androgen deprivation therapy; ADL, activities of daily living; ARPI, androgen receptor pathway inhibitor; CCI, Charlson Comorbidity Index; CGA, comprehensive geriatric assessment; CIRS-G, chronic illness rating scale-geriatric; ECOG, Eastern Cooperative Oncology Group; IADL, instrumental activities of daily living; PSMA, prostate-specific membrane antigen.

**Table 4:** Checklist for the management of [^177^Lu]Lu-PSMA-617 radioligand therapy in older men with castration-resistant metastatic prostate cancer.

Prior to treatment commencement:
• Confirm PSMA expression • Assess disease extent • Assess life expectancy • Assess health status using G8 and “Mini-COG™ • Perform full comprehensive geriatric assessment in patients with G8 score 14/17
When in treatment:
• Monitor haematological reserve (complete blood counts, absolute neutrophil count, platelet count), kidney (creatinine, calculated creatinine clearance) and liver (ALT, AST, ALP, amylase, lipase, total albumin and bilirubin) function • Withhold the dose until improvement to grade 1 or baseline if signs of toxicity including: o grade 2 myelosuppression o grade 2 creatinine increase o spinal cord compression o fractures in weight-bearing bones o grade ≥ 3 fatigue o grade ≥ 2 electrolyte or metabolic abnormalities o 2 non-haematological toxicity • Permanently discontinue if no improvement in toxicity in > 4 weeks • Reduce the dose by 20% in case of: o grade 3 dry mouth o grade ≥ 3 gastrointestinal toxicity o grade ≥ 3 myelosuppression o renal toxicity (> 40% increase in creatinine levels) • Favour the inpatient setting in patients who require close monitoring due to their health status or psychological and social support needs
After treatment:
• Assess haematological reserve and kidney function at 4-6 weeks after the end of the treatment
